# Public Health Guideline to prevent and control SARS-CoV-2 in schools: development and evaluation: Lisa Maria Pfadenhauer

**DOI:** 10.1093/eurpub/ckac130.225

**Published:** 2022-10-25

**Authors:** L Pfadenhauer, M Rueb, B Strahwald, KJ Wabnitz, M Nothacker, EA Rehfuess

**Affiliations:** 1 IBE, Ludwig-Maximilians University, Munich, Germany; 2 Pettenkofer School of Public Health, Ludwig-Maximilians University, Munich, Germany; 3 AWMF e.V., Arbeitsgemeinschaft der Wissenschaftlichen Medizinischen Fachgesellsch, Berlin, Germany

## Abstract

**Issue/problem:**

In times of high demand for scientific evidence for decision-making on COVID-19 mitigation measures, guidelines can be useful for translating scientific evidence into policy and practice. While guidelines are widely used in medical decision-making, they are novel to public health in Germany.

**Description of the problem:**

Since December 2020, a guideline group has been working on a living, evidence- and consensus-based public health guideline on preventing and controlling SARS-CoV-2 transmission in schools. The group includes scientists across multiple disciplines as well as a broad range of stakeholders, including from the school family. Key features in the development of recommendations included a Cochrane rapid review and the WHO-INTEGRATE evidence-to-decision framework. The development and usefulness of the guideline for decision-making are being evaluated using a multi-method approach.

**Results:**

The first version of the guideline containing nine recommendations was published in February 2021. The WHO-INTEGRATE framework facilitated the consideration of factors such as health benefits and harms, feasibility, acceptability and financial constraints. Preliminary findings of the evaluation suggest that under time pressure, developing few essential, consensus-based recommendations while assessing their societal implications is warranted. A shared understanding of evidence and of the purpose and limitations of guidelines is critical. To remain relevant, continuous integration of new evidence and updating of the guideline is necessary.

**Key messages:**

- Purpose and limitation of guidelines needs to be clear to ensure engagement and utilization.
- To remain useful for decision-making, guidelines and the underlying evidence need to be continuously updated.

